# The perils of laboratory research

**DOI:** 10.4103/1817-1737.78435

**Published:** 2011

**Authors:** Feisal A. Al-Kassimi, Muhammad A. Chishi

**Affiliations:** *Department of Medical Biochemistry, King Saud University, Riyadh, Saudi Arabia*; 1 *Department of Medical Biochemistry, King Saud University, Riyadh, Saudi Arabia. Email: falkassimi@yahoo.com*

Sir,

We read with great excitement your September 2010 publication titled “Reactive oxygen metabolites can be used to differentiate malignant and non-malignant pleural effusions”.[1] Cobanoglu *et al*. reported that the level of reactive oxygen molecules (ROMs) are hugely raised in malignant effusions as opposed to no or modest rise in nonmalignant effusions (parapneumonic, tuberculous, and empyema). ROM in serum and pleura fluid differentiated the two categories with a 100% sensitivity and specificity (high in malignant and low in inflammatory effusions). We would like to raise the following concerns:

1. At the time the various criteria (using multiple chemical markers) did not achieve 100% accuracy in distinguishing transudative and exudative effusions, here we have a single test that 100% sensitive and specific in differentiating the etiology of exudates.
2. The Introduction and Discussion sections downplay the importance of ROM in inflammatory conditions and highlight their association with malignancy. As a matter of fact, ROM (including hydrogen peroxide, superoxide anion, and hydroxyl radicals) and also called free oxygen radicals are abundantly produced by polymorphs and other phagocytes to enhance their bactericidal action. Moreover, death in pneumonia was attributed to imbalance (excess) of ROM and lung injury.[2] Significant rises were documented in community-acquired pneumonia in both lung tissue and plasma.[3] Synovial fluid showed enhancement of free radicals in knee arthritis.[4] Serum markers of free radicals were elevated in all 17 patients with tuberculous pleurisy.[5] In the publication in question although the average level of ROM in the serum and pleural fluid of pyogenic and tuberculous pleurisy was significantly higher than controls, 19/30 of serum samples had normal or low levels, and 17/30 of pleural fluid samples had normal or low levels as judged by the reference level in healthy Italians supplied by the manufacturer of the ROM kit used in the study (Diacron, Grosseto, Italy). Do the authors have an explanation for such low levels in inflammatory effusions?
3. We agree with the authors that ROM levels are generally raised in malignant pleural effusions. Studies showed that the elevated levels of ROM differentiated exudative and transudative effusions with excellent sensitivity and specificity. They also contradict the authors’ findings that the ROM level distinguishes malignant and inflammatory effusions. In the best of these studies, published in *Chest* in 2005, the mean and distribution of ROM were the same in malignant and inflammatory effusions.[6] However, the authors of your publication list the above-mentioned study in a different context. Shouldn’t the authors have commented that their findings are different from other published data especially those in *Chest*. Nitrous oxide (NO) is not a ROM but is closely related and enhances their action. NO levels in pleural fluid also failed to differentiate inflammatory and malignant effusions as it was raised in both.[7]
4. The method used by the authors to estimate ROM may throw a light on the discrepancy of results. ROMs are unstable products at room temperature and immediate freezing is mandatory. The storage temperature varies according to the duration of storage. Immediate chilling at –4°C is needed for a few hours storage, but much colder snap freezing is required for long storage. In the publication in your journal we read “pleural fluid, blood samples were kept at room temperature for 30–60 minutes and centrifuged for 10–15 min. Thereafter, fluids were separated from the shaped elements using an automatic pipette. The sera and pleural fluid were kept in the deep freezer at –35°C….” Deep freezers take some time to freeze samples. The considerable delay may explain why inflammatory samples, which are highly cellular and may contain bacteria, had low levels of ROM. The degradation of ROM (especially hydrogen peroxide) is enhanced by contact with high levels of organic matter. These methods compare very unfavorably with other studies where pleural fluid samples were analyzed immediately or lung samples were snap frozen in liquid nitrogen and stored at –80°C.[2,6]
